# Treatment of posttraumatic syringomyelia: evidence from a systematic review

**DOI:** 10.1007/s00701-020-04529-w

**Published:** 2020-08-20

**Authors:** Andrea Kleindienst, Francisco Marin Laut, Verena Roeckelein, Michael Buchfelder, Frank Dodoo-Schittko

**Affiliations:** 1 Department of Neurosurgery, Friedrich-Alexander-University Erlangen-Nurnberg, Erlangen, Germany; 2 Department of Spine Surgery, Krankenhaus Rummelsberg, Schwarzenbruck, Germany; 3 Department of Neurosurgery, University Hospital Puerta de Mar, Cadiz, Spain; 4 Institute of Social Medicine and Health Systems Research, Otto von Guericke University Magdeburg, Magdeburg, Germany

**Keywords:** Syringomyelia, Trauma, Hydromyelia, Treatment, Etiology, Spinal cord injury

## Abstract

**Background:**

Following spinal cord injury (SCI), the routine use of magnetic resonance imaging (MRI) resulted in an incremental diagnosis of posttraumatic syringomyelia (PTS). However, facing four decades of preferred surgical treatment of PTS, no clear consensus on the recommended treatment exists. We review the literature on PTS regarding therapeutic strategies, outcomes, and complications.

**Methods:**

We performed a systematic bibliographic search on (“spinal cord injuries” [Mesh] AND “syringomyelia” [Mesh]). English language literature published between 1980 and 2020 was gathered, and case reports and articles examining syrinx due to other causes were excluded. The type of study, interval injury to symptoms, severity and level of injury, therapeutic procedure, duration of follow-up, complications, and outcome were recorded.

**Results:**

Forty-three observational studies including 1803 individuals met the eligibility criteria. The time interval from SCI to the diagnosis of PTS varied between 42 and 264 months. Eighty-nine percent of patients were treated surgically (*n* = 1605) with a complication rate of 26%. Symptoms improved in 43% of patients postoperatively and in 2% treated conservatively. Stable disease was documented in 50% of patients postoperatively and in 88% treated conservatively. The percentage of deterioration was similar (surgery 16%, 0.8% dead; conservative 10%). Detailed analysis of surgical outcome with regard to symptoms revealed that pain, motor, and sensory function could be improved in 43 to 55% of patients while motor function deteriorated in around 25%. The preferred methods of surgery were arachnoid lysis (48%) and syrinx drainage (31%).

**Conclusion:**

Even diagnosing PTS early in its evolution with MRI, to date, no satisfactory standard treatment exists, and the present literature review shows similar outcomes, regardless of the treatment modality. Therefore, PTS remains a neurosurgical challenge. Additional research is required using appropriate study designs for improving treatment options.

## Introduction

Over the past decades, due to the more frequent routine use of magnetic resonance imaging (MRI) in the diagnostic process and follow-up for back pain and spine injuries, even distinctive features are increasingly detected. The attribution of a T2-hyperintense medullary signal as a prominent central canal, hydro- and syringomyelia has been classified by Milhorat [40, 41]. However, diagnostic criteria and terminology are used inconsistently. Batnitzky [6] differentiated primary congenital and secondary acquired forms of syringomyelia due to trauma, infection, tumor, or vascular disturbances. The pathophysiological mechanism of congenital syringomyelia is explained by the absence of a perforation of the rhombic roof and formation of the foramen Magendie during fetal weeks 6 to 8 [21], resulting in a persistent patent central canal. Conversely, acquired syringomyelia has been linked to intermittent sharp increases in cerebrospinal fluid (CSF) pressure associated with venous pressure fluctuations as the underlying distending force [61]. An experimental modeling of a phase difference between the pressure pulse in the spinal subarachnoid space and the perivascular spaces suggests that mechanical perturbations caused by arachnoiditis exacerbate the phase-lag effect [15]. As soon as the intrinsic fluid storage capacity of the spinal cord is overloaded, medullary edema may develop, presenting as a hyperintense T2-weighted signal and referred to as the “pre-syrinx” state [20].

Following spinal cord injury (SCI), local ischemia, liquefaction of hematoma, and/or autolytic processes, as well as subarachnoid scars limit the CSF flow [17], thereby rendering the development of possible posttraumatic syringomyelia (PTS). In PTS, delayed progressive myelopathy develops often corresponding to spinal segments distant from the level of the original lesion. Besides CSF flow disturbances, posttraumatic kyphosis and the resulting spinal canal stenosis may promote the progression of PTS [1, 44].

Concerning preferred therapeutic strategies, in 2010, a consensus panel gave a strong recommendation for surgical intervention in the setting of motor neurological deterioration and a weak recommendation for spinal cord untethering with expansive duraplasty as the preferred first-line surgical technique [7]. Furthermore, they recommended no decompression at the time of initial injury to limit the future risk of syringomyelia, or for patients developing pain, sensory loss or for asymptomatic but expanding syrinx [7]. While for cervical spondylotic myelopathy, two randomized controlled trials (RCT) compare different surgical techniques [19, 22], no RCT, or, at least, a prospective observational study compares a non-operative to surgical treatment—neither in cervical myelopathy nor in PTS. Here, we present the results of a systematic literature search on PTS for treatment strategy, outcome, and complications.

## Methods

### Eligibility

All studies describing the treatment or reporting the effects of treatment of PTS from 1980 to 2020 were included in this review. Case reports including less than three individuals and animal studies were not included. Apart from this, no restrictions were placed on the study type (experimental or observational studies), or sample size.

### Literature search and data extraction

The retrieval of studies was performed in PubMed using the combined filter and Medical Subject Headings (MeSH) term: (“spinal cord injuries” [Mesh] AND “syringomyelia” [Mesh]). Additionally, the Cochrane Central Register of Controlled Trials (CENTRAL), the Cochrane Database of Systematic Reviews, Web of Science, Scopus, and Google Scholar were searched for eligible studies. All records were screened based on title and abstract independently by two authors (FDS and AK) separately. In cases of discordance, the records were included in the full-text screening. Finally, the remaining records were evaluated by reading the full-text papers. All relevant characteristics (study type, sample size, level and severity of the injury, interval injury to symptoms, surgical technique, follow-up period, main findings) reported in the manuscripts were extracted into evidence tables. Due to a high level of heterogeneity of the studies and many insufficient study designs, no pooled effect estimates were calculated. Instead, a descriptive summary was carried out.

### Risk of bias assessment

Since the Newcastle-Ottawa Scale (NOS) is only suitable for assessing the quality of case-control and prospective cohort studies, we used a risk-of-bias measurement instrument based on the NOS but also suitable for studies which cannot be subsumed under these gold standard observational study designs. A detailed description of this tool has been published by Dodoo-Schittko et al. [12]. All included studies were evaluated by two authors (AK and FDS). Subsequently, disparate ratings were discussed until consensus was reached.

## Results

### Systematic literature search

**Fig. 1:**
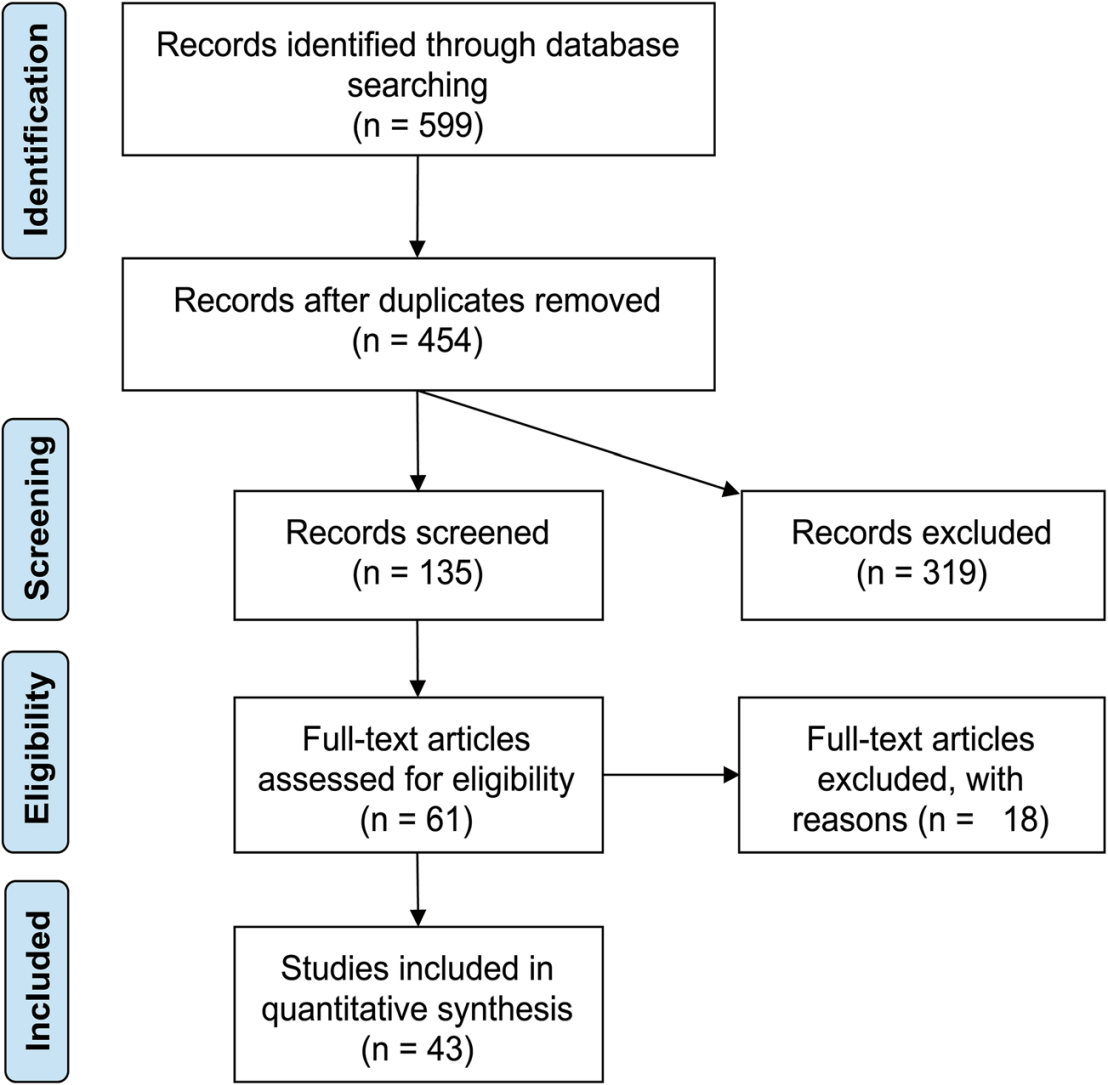
PRISMA flow diagram of the screening process

The electronic search revealed 599 scientific reports, and the PRISMA flow diagram of the screening process is presented in Fig. 1. After screening the title and abstract, 61 articles were included in the full-text screening process. One study was excluded because of overlapping data [54], and one because the full text was not available in English [10]. Finally, a total of 43 studies met the eligibility criteria. The extracted information of these studies is shown in Table 1. All cohorts/samples were independent, and we could not identify any overlap of included individuals. Overall, the sample sizes of the clinical studies detected by literature search ranged from case series of three up to studies including 600 individuals [2, 4, 5, 9, 11, 13, 14, 16, 18, 23–40, 42–53, 55, 56, 59, 60, 62]. Observational designs were applied in all studies. One study used an experimental design investigating the effect of shunt insertion on syrinx length and size in an animal model [8] and an observational study in dogs [3]. Both were excluded because they were not including human subjects.

**Table 1:** Evidence table of the literature search

Author [ref]; study type; sample size	Interval injury symptoms	Severity of spinal cord injury	Level of syrinx	Surgical technique	Follow-up	Findings	Complications of surgery	Conclusions of authors
Shannon [50]; observational; *N* = 13	107 months	54% incomplete; 46% complete	13% cervical; 56% thoracic; 31% lumbar	100% surgery—77% syringostomy and 23% cord transection	18 months	77% complete relief of severe pain was main symptom	n.a.	Syringostomy relieves pain, has a low morbidity, but does not alter sensory symptoms
Vernon [58]; observational; *N* = 27	101 months	46% incomplete; 54% complete	14% cervical; 69% thoracic; 17% lumbar	100% surgery—22% syringostomy, 37% syringo-subarachnoid drain, 22% syringo-peritoneal drain, and 19% cord transsection	60 months	44% improved; 15% stable; 41% deteriorated	29% complication—19% shunt dysfunction, 7% CSF leak, and 4% wound complication	Remissions occur up to 1–5 years; surgery improves symptoms not always, 2 patients deteriorated postop
Rossier [45]; oberservational; *N* = 30	108 months	60% complete; 40% incomplete	100% cervical	63% conservative (*N* = 19); 37% syringo-subarachnoid drain (*N* = 11)	n.a.	Conservative—32% stable and 68% deteriorated; surgery—73% improved	45% complication—18% early neurological deterioration and 27% late neurological deterioration	Some symptoms in conservatively treated patients remained stable over a number of years
Suzuki [51]; observational; *N* = 17	72 months	n.a.	n.a.	100% syringo-peritoneal drain	12 months	82% improved; 18% stable	17% shunt dysfunction	Surgery simple and effective if disease not too advanced
Anton [4]; observational; *N* = 9	68 months	22% incomplete; 78% complete	34% cervical; 54% thoracic; 12% lumbar	33% conservative (*N* = 3); 67% syringo-peritoneal drain (*N* = 6)	n.a.	Conservative—100% stable; surgery—68% improved, 16% stable, and 16% dead	16% shunt dysfunction	Ability to perform activities of daily living not changed by surgery
Williams [61]; observational; *N* = 8	99 months	38% incomplete; 62% complete	25% cervical; 63% thoracic; 12% lumbar	100% arachnoid lysis+syringo-pleural drain	n.a.	38% improved; 38% stable; 25% deteriorated	25% shunt dysfunction	Syrinx drain may improve symptoms
Vaquero [57]; observational; *N* = 9	74 months	100% incomplete	n.a.	100% syringo-subarachnoid drain	19 months	45% improved; 55% stable	11% neurological deterioration	Syringo-subarachnoid drain recommended
Lyons [37]; observational; *N* = 14	101 months	43% incomplete; 57% complete	6% medulla oblongata; 59% cervical; 5% thoracic; 15% atrophic cord; 15% no syrinx but abnormal cord	20% conservative (*N* = 3); 80% surgery (*N* = 11)—8% syringostomy, 8% syringo-subarachnoid drain, 8% syringo-peritoneal drain, 50% syringo-cisternal drain, and 18% cord transsection	24 months	Conservative—33% improved, 67% stable; surgery—68% improved, 16% stable, and 16% deteriorated	83% complication—30% shunt dysfunction, 25% wound complication, 8% meningitis, 8% subdural hematoma, and 8% neurological deterioration	Surgery recommended in progressive SM with neurological deterioration; abnormal cord considered precursor of syrinx
La Haye [33]; observational; *N* = 8	96 months	100% incomplete	66% cervical; 34% thoracic	13% conservative (*N* = 1); 87% surgery (*N* = 7)—86% cysto-peritoneal drain without valve	40 months	Conservative—100% stable; surgery—86% improved and 14% deteriorated	n.a.	Cyst drainage by pressure difference
Tator [52]; observational; *N* = 11	83 months	n.a.	n.a.	100% surgery—9% syringostomy, 73% syringo-subarachnoid drain, 9% arachnoid lysis+syringo-subarachnoid drain, and 9% cord transsection	55 months	55% improved; 18% stable; 27% deteriorated	27% shunt dysfunction	Duration of symptoms and neurological deficit correlated to outcome; early surgery warranted in progressive, symptomatic SM
Padovani [42]; observational; *N* = 4	72 months	100% incomplete	n.a.	100% syringo-subarachnoid drain	60 months	50% improved; 50% stable 100% MRI improved	25% neurological deficit	No relationship between duration of symptoms and outcome
Hida [24]; observational; *N* = 14	148 months	36% incomplete; 64% complete	35% cervical; 35% thoracic; 30% lumbar	22% conservative (*N* = 3); 78% surgery (*N* = 11)—54% syringo, subarachnoid drain, 36% syringo-peritoneal drain, and 10% ventriculo-peritoneal shunt	44 months	Conservative, 100% stable; surgery, 100% improved and 100% MRI improved	80% shunt dysfunction	Syringo-subarachnoid drain should be first option
Edgar [12]; observational; *N* = 525	240 months	18% incomplete 82% complete	75% cervical; 24% thoracic; 1% lumbar	100% surgery—syringostomy, syrinx drain, and cord transsection	26 months	87% improved if symptoms < 3 months; 44% improved if symptoms > 6 months 231/525	26% complication—12% shunt dysfunction, 5% neurological deficit (transient), 4% CSF leak, 3% neurological deficit (permanent), 1% wound complication, and 0.4% spine instability	Myelopathy can precede SM; untethering and duraplasty very successful with preference for early intervention
Wiart [59]; observational; *N* = 8	54 months	62% incomplete; 38% complete	62% cervical; 25% thoracic	100% syringo-peritoneal drain	54 months	50% improved; 50% deteriorated; 100% MRI improved	50% neurological deterioration	Syringo-peritoneal drain is efficient in syrinx treatment but does not prevent meningeal fibrosis
Sgouros [49]; observational; *N* = 57	91 months	28% incomplete; 72% complete	23% cervical; 67% thoracic; 10% lumbar	100% surgery—14% arachnoid lysis, 4% syringostomy, 11% syringo-subarachnoid drain, 49% syringo-pleural drain, and 28% cord transection ± drain	90 months	83% stable; 53% of drains effective after 4 years	42% complication—29% drain related (dyslocation, occlusion, broncho-pleural fistula, infection), 4% wound complication, 3% meningitis, 3% pneumo-cephalus, and 1% CSF leak	Decompressive laminectomy together with reconstruction of subarachnoid space more effective and fewer complications
el Masry [13]; observational; *N* = 26	101 months	32% incomplete; 68% complete	32% cervical; 46% thoracic; 22% lumbar	14% conservative (*N* = 4); 86% surgery (*N* = 22)—5% syringostomy, 30% syringo-subarachnoid drain, 55% syringo-pleural drain 12, and 10% cord transsection	36 months	Conservative—100% stable; surgery—60% improved, 28% stable, and 14% deteriorated	16% complication—8% air embolims, 4% pneumocephalus, and 4% wound complication	No difference of results with regard to level/extend of syrinx or severity of initial injury; no shunt procedure superior
Schurch [48]; pbservational; *N* = 20	112 months	20% incomplete; 80% complete	60% cervical; 40% thoracic	65% conservative (*N* = 13); 35% surgery (N = 7)—29% spinal stabilization, 29% syringostomy, and 42% syringo-subarachnoid drain	70 months	Conservative—77% stable and 23% deteriorated; surgery—72% improved, 14% stable, and 14% deteriorated	n.a.	Close relationship between medullar compression, kyphosis, and neurological deterioration; re-alignment and stabilization can prevent PTS
Asano 1996 [5]; observational; *N* = 9	80 months	n.a.	34% cervical; 66% thoracic	44% conservative (*N* = 4); 56% syringo-subarachnoid drain (*N* = 5)	n.a.	Conservative—100% stable; surgery—100% improved	40% shunt dysfunction	Pre-op MRI may help to identify “high-pressure” syrinx
Kramer [32]; observational; *N* = 17	n.a.	33% incomplete; 67% complete	33% cervical; 66% thoracic	53% conservative (*N* = 9); 47% syringostomy (*N* = 8)	43 months	Conservative—23% improved, 33% stable, and 44% deteriorated; surgery—75% improved and 25% deteriorated	No complication	Pain and sensory deficit respond better to surgery than spasticity
Ronen [44]; observational; *N* = 10	104 months	50% incomplete; 50% complete	50% cervical; 10% thoracic; 40% lumbar	50% conservative (*N* = 5) only rehabilitation; 50% surgery+rehabilitation (*N* = 5)—20% laminectomy and 80% syringo-subarachnoid drain	Conservative—70 months; surgery—66 months	Conservative—20% improved, and 80% stable; Surgery—20% stable and 80% deteriorated	No complication	No clear evidence for the superiority of surgery
Schaller [47]; observational; *N* = 12	146 months	17% incomplete; 83% complete	17% cervical; 75% thoracic; 8% lumbar	100% surgery—17% arachnoid lysis and 83% arachnoid lysis+syringo-peritoneal drain (low pressure)	44 months	n.a.	30% shunt failure	Better results without drain
Hess [23]; observational; *N* = 8	120 months	50% incomplete; 50% complete	63% cervical; 25% thoracic; 22% lumbar	100% surgery—87% syringo-subarachnoid drain and 13% syringo-pleural drain	180 months	87% improved; 13% deteriorated	50% shunt failure; 25% new syrinx	Less pain and improved strength are more significant than decreased numbness
Holly [25]; observational; *N* = 5	24–264 months	20% incomplete; 80% complete	20% cervical; 20% thoracic; 10% lumbar	100% surgery; ventral epidural decompression	38 months	80% improved; 20% stable	n.a.	Anatomical reconstruction of spinal deformities recommended
Lee [38]; observational; *N* = 34	132 months	n.a.	65% cervical; 25% thoracic; 10% lumbar	100% surgery—(A) 41% arachnoid lysis if tethering, (B) 47% syringo-subarachnoid drain if no tethering, and (C) 12% arachnoid lysis+drain if tethering and persistent cyst	29 months	76% improved; 18% stable; 6% deteriorated; 90% MRI improved	32% complication—(A) 7% failure, 14% complication, (B) 13% failure, 13% complication, and (C) 25% CSF leak, 75% neurological deficit (transient)	Arachnoid lysis is effective if tethering and intra-op cyst collapse
Lee 2001 [39]; observational; *N* = 45	78 months	n.a.	62% cervical; 30% thoracic; 8% lumbar	100% surgery—40% arachnoid lysis, 38% syringo-subarachnoid drain, 20% arachnoid lysis+drain, and 2% arachnoid lysis, subsequent drain	23 months	33% improved, 15—60% stable, 27; 7% deteriorated, 3; 93% MRI improved, 42	16% complication—7% shunt failure, 2% CSF leak, and 7% neurological deficit (transient)	Untethering can reduce cyst size and alleviate symptoms in the majority; duraplasty may be more physiological
Schaan [46]; observational; *N* = 30	42 months	20% incomplete; 80% complete	33% cervical; 67% thoracic	100% surgery—(A) drain; (B) arachnoid lysis+drain+duraplasty—73% syringo-subarachnoid drain, 13% syringo-peritoneal drain, and 3% syringo-pleural drain; and (C) arachnoid lysis+duraplasty	(A) 80 months; (B) 52 months; and (C) 46 months	50% improved; 33% stable; 14% deteriorated; 3% dead	1 death caused by pneumonia (3%)	No significant difference for pain, motor deficit, sensory deficit between surgical procedures
Lee [36]; observational; *N* = 3	n.a.	100% incomplete	66% thoracic; 33% holocord	100% surgery—33% arachnoid lysis+duraplasty and 67% arachnoid lysis+syringo-subarachnoid drain	14 months	66% improved; 33% stable	No complication	Restoration of CSF flow by decompression more effective than syrinx drain
Carroll [11]; observational; *N* = 15	70 months	50% incomplete; 50% complete	31% cervical; 56% thoracic; 7% lumbar	6% conservative (*N* = 1); 94% surgery (*N* = 14)—47% syringo, peritoneal drain, 32% syringostomy+syringo-peritoneal drain, 7% arachnoid lysis, and 7% duraplasty	n.a.	Conservative—100% stable; surgery—31% improved, 25% stable, 19% deteriorated, 13% unavailable, and 6% dead	6% dead 1	Surgery has a positive effect on symptom progression, although no recommendation on optimal intervention
Jaksche [28]; observational; *N* = 58	n.a.	n.a.	n.a.	100% surgery—17% drain and 83% arachnoid lysis+duraplasty	120	59% improved; 29% stable; 9% deteriorated;3% dead	80% shunt dysfunction; 3% dead (pulmonary embolism)	Restoration of normal CSF flow reduces shearing force on spinal cord
Laxton [35]; observational; *N* = 4	123 months	50% incomplete; 50% complete	25% cervical; 75% thoracic	100% cord transsection	54 months	100% improved	No complication	Cord transsection should be avoided in incomplete SCI
Lam [34]; observational; *N* = 3	n.a.	100% incomplete	67% cervical; 33% thoracic	100% subarachnoid-peritoneal drain—level C4 for C0-Th1 and level Th4 below Th1	33 months	100% improved	67% complication—33% shunt dysfunction and 33% cerebellar tonsillar descent	Risk of cerebellar tonsillar descent
Cacciola [9]; observational; *N* = 8	n.a.	n.a.	13% cervical; 63% thoracic; 13% lumbar; 13% holocord	100% syringo-pleural drain	38 months	50% improved; 24% stable; 13% deteriorated; 13% dead	20% postmyelotomy pain; 13% dead	Causal surgery should be performed first; shunt placement is second-line option
Falci [17]; observational; *N* = 362	128 months	63% ASIA (A); 10% ASIA (B); 11% ASIA (C) 14% ASIA (D); 1% ASIA (E)	68% cervical; 32% thoracic	100% surgery—80% arachnoid lysis+duraplasty and 20% syringo-subarachnoid/peritoneal drain	144 months	59% improved spasticity—90% stable and 0.5% dead	7% complication—4% CSF leak, 1% deep venous thrombosis, 1% pulmonary embolism, 0.5% meningitis, 0.5% wound complication, 0.5% dead, and 0.2% myocardial infarction	No significant change ASIA pre- and postop; surgery recommended in progressive myelopathy
Ushewokunze [54]; observational; *N* = 40	72 months	40% incomplete; 60% complete	n.a.	100% duraplasty—43% additional procedures (29% revision of duraplasty, 76% lumbo/ventriculo-peritoneal shunt, 6% syringostomy, 35% syringo-subarachnoid/pleural/peritoneal drain, 6% percutaneous syrinx aspiration)	64 months	68% stable; 32% deteriorated; 23% MRI improved at 6 months	43% complication—13% dysaesthic pain, 10% neurological deficit, 10% wound complication, 5% CSF leak, 3% posterior fossa subdural haematoma, and 3% hydrocephalus	Decompression and arachnoid lysis have limited effect on the long-term symptoms
Ewelt [15]; observational; *N* = 15	n.a.	53% incomplete; 47% complete	6 levels (range 1–16)	100% arachnoid lysis+cord transsection	24 months	40% improved; 53% stable; 7% deteriorated	No complication	Cord transsection alternative option for progressive SM and adhesive arachnoitis
Oluigbo [41]; observational; *N* = 5	n.a.	100% incomplete	40% cervical; 60% thoracic	100% surgery—80% decompression+lumbo-peritoneal shunt and 20% lumbo-peritoneal shunt	25 months	40% improved; 60% deteriorated; 60% MRI improved	60% shunt revision	Lumbo-peritoneal drain indicated if no CSF obstruction visible
Aghakhani [2]; observational; *N* = 34	133 months	100% incomplete	9% cervical; 70% thoracic; 21% lumbar	100% surgery—(A) 56% arachnoid lysis and (B) 44% drain	(A) 84 months; (B) 46 months	(A) 73% improved, 21% stable, and 5% deteriorated; (B) 47% stable and 53% deteriorated	68% complication—53% shunt revision, 9% CSF leak, 3% meningitis, and 3% pneumonia	Early correction of spinal canal stenosis essential; subarachnoid space reconstruction and cyst opening is safe and effective
Klekamp [31]; observational; *N* = 137	135 months	33% ASIA (A + B); 0% ASIA (C + D); 27% ASIA (E)	22% cervical; 66% thoracic; 12% lumbar	55% conservative (*N* = 76); 45% surgery (*N* = 61)—88% arachnoid lysis+duraplasty and 3% cord transection	Conservative—67 months; surgery n.a.	Conservative—67% stable; Surgery*:* ASIA (A + B)—65% improved and 35% stable; ASIA (C + D)—52% improved, 39% stable, and 9% deteriorated; ASIA E—38% improved, 50% stable, 13% deteriorated	16% complication, 13% revision, 8% neurological deficit (transient), 5% wound infection, 5% hematoma, 2% CSF leak, 2% cardiac arrest, 22% 5-year recurrence, and 56% 10-year recurrence	Decompression with arachnolysis, untethering, and duraplasty provides good long-term results for patients with progressive neurological symptoms; Treatment of patients with preserved motor functions remains a major challenge
Isik [27]; *N* = 19	24 months	n.a.	n.a.	100% surgery—11% syringostomy, 26% syringo-subarachnoid drain, and 63% syringo-pleural drain	108 months	82% improved; 6% stable; 12% deteriorated; 100% MRI improved	47% complication—20% neurological deficit (transient), 2% drain dislocation, 12% revision, and 6% neurological deficit (permanent)	Syringo-pleural shunt produced satisfactory results at long-term follow-up
Hayashi [22]; observational; *N* = 20	126 months	45% incomplete; 55% complete	20% cervical; 55% thoracic; 25% lumbar	100% arachnoid lysis+syringo-subarachnoid drain	48 months	60% improved; 20% stable; 20% deteriorated	No complication	No correlation pre- and postop ASIA; correlation clinical outcome and syrinx size
Kim [30]; observational; *N* = 9	264 months	100% incomplete	67% cervical; 33% thoracic	100% surgery—33% syringo-subarachnoid drain, 44% arachnoid lysis+duraplasty, and 22% syringo-pleural drain	112 months	11% improved; 44% stable; 44% deteriorated	33% complication—22% shunt dysfunction and 11% wound complication	Unfavorable long-term outcome with surgery
Karam [29]; observational; *N* = 27	144 months	52% ASIA (A); 11% ASIA (C); 37% ASIA (D)	15% cervical; 78% thoracic; 7% lumbar	100% surgery—60% drain (12% syringo-pleural, 88% syringo-subarachnoid), 25% arachnoid lysis+syringo-pleural drain+duraplasty, and 15% arachnoid lysis+duraplasty	216 months	52% improved; 37% stable; 11% deteriorated	62% revision of drain; 27% revision of duraplasty	Duraplasty and arachnoid lysis preferred over drain
Holmstrom [26]; observational; *N* = 17	n.a.	n.a.	53% cervical; 41% thoracic; 6% lumbar	100% arachnoid lysis+syringo-subarachnoid drain	n.a.	50% improved; 31% stable; 19% deteriorated (3/16); 66% MRI improved (6/9)	n.a.	Untethering and cyst drainage resulted in patient satisfaction

*n.a.*, not available; *CSF*, cerebrospinal fluid; *SM*, syringomyelia; *PTS*, posttraumatic syringomyelia; *MRI*, magnetic resonance imaging; *SCI*, spinal cord injury; *ASIA*, American Spinal Injury Association

### Patients characteristics

The studies collect data from 1803 patients. The time interval from the spinal injury to the onset of symptoms varies between 42 and 264 months. Pain, motor, and sensory function compromise are the most frequent symptoms, while autonomic dysfunction is uncommon. Complete SCI preponderates (mean 63%) over incomplete (mean 37%). The most frequent locations of spinal injuries were the thoracic region (mean 48%) and the cervical region (mean 41%). The lumbar region (mean 10%) and medulla oblongata (mean < 1%) were hardly affected.

### Clinical outcome

The mean follow-up period was 56 months in the observational studies. While 89% of patients were treated surgically, of the remaining 11% treated conservatively, only those of Ronen et al. [45] received a rehabilitation (*n* = 5). The summarized results of the surgical and conservative treatment are presented in Table 2. Following surgery for PTS, 43% of patients improved clinically, and the MRI findings in 75% of patients; 50% remained stable, 16% deteriorated, and 0.8% died peri-procedural. Sufficient information about complications of the performed surgical procedures is not available in all studies. Six studies do not describe complications, and in another six studies, no complications were observed. Out of the 403 complications following surgery (26%), a drain or valve dysfunction was most often reported (21%). A CSF leak was reported in 2.9%, a transient neurological deficit in 2.8%, a permanent neurological deficit in 2.5%, and wound complications were reported in 2% of patients. Other complications included venous thromboembolic event (*N* = 7), meningitis (*N* = 6), pneumocephalus (*N* = 3), air embolism (*N* = 2), cerebellar tonsillar descent (*N* = 1), and cardiac arrest (*N* = 1).

**Table 2:** Results of surgical and conservative treatment in posttraumatic syringomyelia

Detailed results of treatment	Surgery (*N* = 1605)	Conservative (*N* = 198)
MRI improved	123/164 (75%)	n.a.
Improved	510/1175 (43%)	4 (2%)
Stable	585/1078 (50%)	174 (88%)
Deteriorated	108/659 (16%)	20 (10%)
Dead	8/1021 (0.8%)	n.a.
Complications	403/1561 (26%)	n.a.
Drain or valve dysfunction	207/973 (21%)	
CSF leak	46/1561 (2.9%)	
Transient neurological deficit	44/1561 (2.8%)	
Permanent neurological deficit	39/1561 (2.5%)	
Wound complication	32/1561 (2.0%)	
Other		
Venous thromboembolic events	7	
Meningitis	6	
Pneumencephalus	3	
Subdural hematoma	2	
Air embolism	2	
Cerebellar tonsillar descent	1	
Cardiac arrest	1	

It is important to note that the comparison of surgical and conservative treatment lacks a baseline, which carries the risk of selection bias per chosen treatment

MRI, magnetic resonance imaging; CSF, cerebrospinal fluid

A detailed analysis of surgical results concerning pain, sensory, motor, and autonomic function was performed by Vernon et al. in 27 patients [59], Lee et al. in 87 patients [37, 38], and Schaan et al. in 30 patients [47]. The results are presented in Table 3. Pain is improved in 43% of patients, sensory function in 49%, and motor function in 55%. On the other hand, pain is deteriorated in 15%, sensory function in 27%, motor function in 25%, and autonomic function in almost all cases.

**Table 3:** Detailed analysis of surgical results concerning pain, sensory, motor, and autonomic function

Results of treatment	Pain	Sensory function	Motor function	Autonomic dysfunction
Improved total	46/106 (43%)	42/85 (49%)	50/91 (55%)	2/15 (13%)
Vernon [54] (total *N* = 27)				
Syringostomy (*N* = 3)	3/3	0/2	1/3	0/1
Syringostomy + drain (*N* = 16)	8/14	7/13	9/13	–
Cord incision/transection (*N* = 8)	4/6	3/6	4/4	0/2
Lee [35] (total *N* = 34)				
Arachnoid lysis (*N* = 14)	4/12	3/6	4/7	1/3
Syringo-subarachnoid drain (*N* = 16)	5/13	3/6	5/10	0/2
Arachnoid lysis + drain (*N* = 4)	1/4	1/3	2/3	–
Lee [34] (total *N* = 53)				
Arachnoid lysis (*N* = 19)	6/15	4/9	6/10	1/4
Syringo-subarachnoid drain (*N* = 17)	5/13	4/7	6/11	0/2
Arachnoid lysis + drain (*N* = 9)	2/8	2/6	3/6	0/1
Schaan [42] (total *N* = 30)				
Drain procedures (*N* = 18)	5/14	5/15	5/13	–
Drain + duraplasty (*N* = 5)	1/1	4/5	3/5	–
Duraplasty (*N* = 7)	3/3	6/7	2/6	–
Stable total	11/41 (27%)	10/48 (21%)	9/44 (20%)	0/3 (0%)
Vernon 1983 [54] (total *N* = 27)				
Syringostomy (*N* = 3)	0/3	0/2	1/3	0/1
Syringostomie + drain (*N* = 16)	4/14	1/13	0/13	–
Cord incision/transection (*N* = 8)	2/6	0/6	0/4	0/2
Schaan [42] (total *N* = 30)				
Drain procedures (*N* = 18)	5/14	8/15	4/13	–
Drain + duraplasty (*N* = 5)	0/1	0/5	1/5	–
Duraplasty (*N* = 7)	0/3	1/7	3/6	–
Deteriorated total	6/41 (15%)	13/48 (27%)	11/44 (25%)	3/3 (100%)
Vernon [54] (total *N* = 27)				
Syringostomy (*N* = 3)	0/3	2/2	1/3	1/1
Syringostomie + drain (*N* = 16)	2/14	5/13	4/13	–
Cord incision/transection (*N* = 8)	0/6	3/6	0/4	2/2
Schaan [42] (total *N* = 30)				
Drain procedures (*N* = 18)	4/14	2/15	4/13	–
Drain + duraplasty (*N* = 5)	0/1	1/5	1/5	–
Duraplasty (*N* = 7)	0/3	0/7	1/6	–

The type of surgical procedure on PTS was specified in 866 patients and is presented in Table 4. Arachnoid lysis was the procedure that was performed most often (*N* = 418; 48%), followed by various techniques of drain placement (*N* = 267; 31%). Procedures that were performed less frequently were cord transection (*N* = 51; 5.9%), syringostomy (*N* = 49; 5.7%), duraplasty (*N* = 41; 4.7%), a combination of arachnoid lysis and drain (*N* = 30; 3.5%), decompression alone (*N* = 5; 0.6%), or shunt alone (*N* = 5; 0.6%).

**Table 4:** Detailed analysis of results of different surgical techniques in the treatment of posttraumatic syringomyelia

Methods of surgical treatment (*N* = 866)	Improved	Stable	Deter.	Complications
Arachnoid lysis (*N* = 418 (48%))				
Lee [35] (*N* = 14)	Pain 33% (4/12)			Failure 7% (1/14)
	Sensory 50% (3/6)			Neurol. deficit 14% (2/14)
	Motor 57% (4/7)			CSF leak 7% (1/14)
Lee [34] (*N* = 19)	Pain 40% (6/15)			Failure 5% (1/19)
	Sensory 44% (4/9)			Neurol. deficit 11% (2/19)
	Motor 60% (6/10)			CSF leak 5% (4/19)
Aghakhani [2] (*N* = 19)	Postop 16% (3/19)	79% (15/19)	5% (1/19)	
Drain (syringo-subarachnoid/pleural/peritoneal) (*N* = 267 (31%))				
Lee [35] (*N* = 16)	Pain 38% (5/13)			Failure 13% (2/16)
	Sensory 50% (3/6)			Trans. neurol. deficit 13% (2/16)
	Motor 50% (5/10)			
Lee [34] (*N* = 17)	Pain 38% (5/13)			Failure 18% (3/17)
	Sensory 57% (4/7)			Trans. neurol. deficit 5% (1/17)
	Motor 54% (6/11)			
Schaan [42] (*N* = 18)	Pain 36% (5/14)	36% (5/14)	29% (4/14)	
	Sensory 33% (5/15)	53% (8/15)	13% (2/15)	
	Motor 38% (5/13)	31% (4/13)	31% (4/13)	
Aghakhani [2] (*N* = 15)	Postop 0% (0/15)	47% (7/15)	53% (8/15)	
Cord transection (*N* = 51 (5.9%))				
Vernon [54] (*N* = 5)	Pain 75% (3/4)	25% (1/4)	0% (0/4)	
	Sensory 40% (2/5)	0% (0/5)	60% (3/5)	
	Motor 100% (3/3)	0% (0/3)	0% (0/3)	
Syringostomy (*N* = 49 (5.7%))				
Vernon [54] (*N* = 22)	Pain 63% (12/19)	26% (5/19)	11% (2/19)	
	Sensory 31% (5/16)	6% (1/16)	69% (11/16)	
	Motor 59% (10/17)	0% (0/17)	41% (7/17)	
Duraplasty (*N* = 41 (4.7%))				
Schaan [42] (*N* = 12)	Pain 100% (4/4)	0% (0/4)	0% (0/4)	
	Sensory 83% (10/12)	8% (1/12)	8% (1/12)	
	Motor 45% (5/11)	36% (4/11)	18% (2/11)	
Arachnoid lysis + syringo-subarachnoid drain (*N* = 30 (3.5%))				
Lee [35] (*N* = 4)	Pain 25% (1/4)			
	Sensory 67% (2/3)			Trans. neurol. deficit 75% (3/4)
	Motor 67% (2/3)			CSF leak 25% (1/4)
Lee [34] (*N* = 9)	Pain 25% (2/8)			Failure 33% (3/9)
	Sensory 33% (2/6)			Trans. neurol. deficit 67% (6/9)
	Motor 50% (3/6)			CSF leak 11% (1/9)

Decompression (*N* = 5 (0.58%)). Ventriculo/lumbo-peritoneal shunt (*N* = 5 (0.58%))

*Deter.*, deterioration; *Neurol.*, neurological; *CSF*, cerebrospinal fluid; *Trans.*, transient

Four studies directly compared the results of different treatment regimes in separate cohorts. The allocation process was either based on clinical findings [37, 38] or not described [2, 47], and the time point of outcome assessment is not always specified. In two separate studies, Lee et al. performed arachnoid lysis (*N* = 33), drain placement (*N* = 33), and a combination of both (*N* = 13). While the failure of drain placement (*N* = 5; 15%) is higher than of arachnoid lysis (*N* = 2; 6%), the incidence of procedure-related neurological deficits (9%: arachnoid lysis *N* = 3; drain *N* = 3) and the overall improvement is comparable (pain 30%: each *N* = 11; sensory 21%: each *N* = 7; motor 30–33% arachnoid lysis *N* = 10, drain *N* = 11). The results reported by Aghakhani et al. postoperatively were worse for drain placement (*N* = 15; improvement 0%; deterioration 53%) than for arachnolysis (*N* = 19: improvement 16%; deterioration 5%) but improved over time [2]. A combination of both techniques was performed in a minority of patients and was associated with a considerable morbidity (53–75%) [2, 37, 38]. Schaan et al. compared drain placement (*N* = 18: improvement 33–38%; deterioration 13–31%) and duraplasty (*N* = 12; improvement 45–100%; deterioration 0–18%) [47].

The results of the less-often performed procedures are better, although based on small sample sizes: cord incision or transection resulted in an improvement of 50 to 100% (*N* = 8) [58], syringostomy resulted in an improvement of 47% to 71% (*N* = 19) [58], and duraplasty resulted in an improvement of 33% to 100% (*N* = 12) [47].

Without a surgical treatment—i.e., following a “conservative” treatment—2% of patients improved and 88% remained stable, while 10% of patients deteriorated (total *N* = 198; Table 2) [4, 5, 11, 14, 25, 32–34, 40, 45, 46, 49].

One experimental study including six patients (male, 30–50 years) into a phase 2 trial injecting autologous mesenchymal stromal cells into the syrinx of PTS patients was not included in the above-mentioned analysis because it is a novel therapeutic approach [57]. The time interval between SCI and treatment varied from 5.8 to 27.7 years, and irrespective of syrinx size, 300 × 10^6^ autologous expanded mesenchymal stromal cells, supported in autologous plasma were administered into the syrinx. The patients were followed up for 6 months, and the authors report in all patients variable improvement in clinical scales, mainly in the scales related to sphincter dysfunction and neuropathic pain [57].

## Discussion

Over the past four decades, PTS is diagnosed more frequently and surgical techniques became more elaborate. Consequently, in a review paper (2010), Bonfield et al. presented recommendations of a consensus panel for surgical intervention in the setting of motor neurological deterioration and for spinal cord untethering with duraplasty as the preferred surgical technique [7]. Interestingly, they recommended against the direct decompression at the time of initial injury as well as against surgical interventions for patients developing pain, sensory loss, or for asymptomatic but radiologically expanding syrinx [7]. By 2020, still, no prospective study is available comparing non-operative and surgical treatment—neither in cervical myelopathy nor in PTS.

Here, we present the results of a systematic bibliographic literature search on PTS for treatment strategy, outcome, and complications. The risk of bias assessment revealed a high or unclear risk of selection bias in all studies. In addition, the risk of information bias was present in many studies, notably in the assessment of patient-reported outcome measurements. This is because of the use of non-validated measurement instruments. The present literature review reveals that 89% of the included 1803 patients were treated surgically. This fact can be probably attributed to a publication bias. Nevertheless, 12 studies including 198 PTS patients treated conservatively have been published [4, 5, 11, 14, 25, 32–34, 40, 45, 46, 49]. In contrast with the expert recommendations of 2010 [7], we also appreciate the results of these conservatively treated PTS patients.

### Effect of intervention on specific symptoms

The 2010 recommendations for surgical intervention in PTS advocate spinal cord untethering with duraplasty in the setting of motor neurological deterioration but against surgery for pain and sensory deterioration [7]. When we analyzed the outcome of surgery for PTS concerning specific neurological functions (Table 3), motor and sensory dysfunction responded better than pain to an intervention (improved motor 55%, sensory 49%, pain 43%) but were also at a higher risk for deterioration (motor 25%, sensory 27%, pain 15%) while the autonomic function was at a high risk for deterioration [37, 38, 47, 59]. Hence, although it is common practice in neurosurgery to prioritize motor symptoms in decision making for surgery and in guidelines, the recommendation by Bonfield et al. do not necessarily reflect the common practice in the existing literature on PTS.

The decision as to if, when, and what type of treatment to offer to a patient with PTS has changed over the past four decades. During the 1980s and early 1990s, surgical intervention including syringostomy and syrinx drainage was the preferred treatment option [58, 60]. Drainage complications resulted in the preference for the reconstruction of the subarachnoid space [50] or even a conservative treatment [5, 14, 33, 45, 49]. Furthermore, one has to keep in mind that both, improvement as well as an arrest of deterioration, may constitute the goal of treatment.

### Effect of type of surgical procedure on the outcome

The type of surgical procedure was specified in almost 900 patients (Table 4). Forty-eight percent of authors performed untethering, another 31% various techniques of drain placement, and 4% combined both procedures. Cord transection, syringostomy, or duraplasty were performed by around 5%, and less than 1% applied decompression or shunt alone. While no surgical technique for PTS provides substantially superior results, any type of drain placement is associated with a failure rate of up to 20% [37, 38, 47]. The reason for the weak recommendation of the 2010 consensus panel for spinal cord untethering with expansive duraplasty as the preferred first-line surgical technique is not obvious [7]. From a pathophysiological point of view, an individualized therapeutic approach would be desirable based on the patient’s history, symptoms, and radiological findings. After the detection of local alterations in CSF flow, the restoration of physiological flow patterns should be the first goal followed by draining CSF trapped in cysts. Duraplasty may aid in the creation of extra CSF space and avoid new scar formation. Cell transplantation therapies in PTS are under investigation, although their relevance has not been confirmed yet [57, 63].

### Time interval to surgical intervention

Experimental evidence indicates that mechanical perturbations of arachnoiditis form the basis of syrinx development [15], and medullary edema in MRI can be interpreted as a “pre-syrinx “[20] as suggested by Lyons et al. in the pre-MRI area [40]. These pathophysiological considerations may stress the relevance of an early intervention to divert CSF if the spinal canal stenosis is not absolute [13, 29, 40, 53]. Irrespectively, it is self-evident that the spinal stabilization and re-alignment of fractures preventing medullar compression and kyphosis is mandatory [2, 44, 49]. Interestingly, the 2010 consensus committee recommended against the direct decompression at the time of initial injury as well as against surgical interventions for patients developing sensory loss, a pain syndrome, or for asymptomatic but expanding syrinx [7].

Some authors do not support the use of a surgical intervention in PTS, even in patients with progressive neurological deterioration [45]. Our analysis suggests that a conservative treatment as reported by some authors [4, 5, 11, 14, 25, 33, 34, 40, 45, 49] may be an alternative to surgical procedures flawed by complications. However, because of the observational design of all studies, we cannot exclude that our analysis is biased by a crossover of patients who were initially treated conservatively and were referred to a subsequent surgical treatment because of clinical deterioration. Furthermore, it is important to keep in mind that scar formation itself without PTS may result in neurological deterioration.

## Conclusion

Here, we present the analysis of a systematic literature search on therapeutic options for PTS over four decades. The outcome of conservative and surgical treatment is not directly comparable because of the exclusively observational study design with the subsequent selection bias and cross-over. While a satisfying outcome defined as either an improved or stable situation is identical (conservative 85%; surgery 88%), the reduction of deterioration from 15.5% without surgery to 9.1% with surgery is accompanied by a 0.33% surgery-related mortality and 23% complications. The evidence of the efficacy of the different treatment modalities is very low mainly resulting from the application of observational study designs with a consistently high risk of selection bias. This points to the necessity of additional research using appropriate study designs to reveal the causal relationship between treatment and outcome. However, concerning the existing literature, there is no satisfactory standard treatment for syringomyelia even diagnosing PTS early in its evolution. Hence, PTS remains a neurosurgical challenge even diagnosed early in its course.

## Abbreviations

PTS: Posttraumatic syringomyelia
SCI: Spinal cord injury
MRI: Magnetic resonance imaging
CT: Computed tomography
CSF: Cerebrospinal fluid
ASIA: American Spinal Injury Association
